# HIV testing during the neonatal period

**DOI:** 10.4102/sajhivmed.v16i1.362

**Published:** 2015-04-24

**Authors:** Gayle G. Sherman

**Affiliations:** 1 Department of Paediatrics and Child Health, University of the Witwatersrand, South Africa; 2 Centre for HIV and STI, National Institute for Communicable Diseases, South Africa

## Introduction

Testing for HIV in the neonatal period has been routinely recommended for all HIV-exposed infants in the developed world for over two decades. In 2015, birth testing for certain asymptomatic HIV-exposed infants was included in the South African National Consolidated Guidelines for the first time.^1^ Questions remain concerning the optimal recommendations for and implementation of HIV testing in neonates to achieve improved outcomes for HIV-infected infants in South African and other low-resource settings.

## Effect of evolving prevention of mother to child transmission interventions on 6-week HIV polymerase chain reaction diagnostic performance

The notion that a single HIV polymerase chain reaction (PCR) test performed at 6 weeks of age would detect virtually all *in utero* and intrapartum HIV-infected infants failed to recognise:

- the HIV-related mortality that occurs prior to testing at 6 weeks of age^2,3,4^
- the reduced sensitivity of HIV PCR tests as a consequence of the increase in the number and duration of drugs used for prevention of mother-to-child transmission of HIV (PMTCT) prophylaxis.

There is increasing evidence that both fixed dose combination (FDC) maternal PMTCT prophylaxis and daily dose nevirapine (NVP) infant prophylaxis (Option B or B+) contribute towards reduced detection of perinatal HIV infection at 6 weeks of age. The literature demonstrates that:

- a single perinatal dose of NVP reduced viral load to below the limit of detection in 38% and 17% of *in utero* infected infants at 5 days and 2 weeks of age respectively.^5,6^ No HIV PCR sensitivity data for 6-week-old HIV-exposed infants, tested at discontinuation of 6 weeks of daily dose NVP, are available.
- the probability of a positive HIV PCR at age 6 weeks in perinatally HIV-infected infants is decreased with multi-drug maternal and/or infant PMTCT prophylaxis^7^
- in non-breastfed infants, HIV DNA and RNA PCR sensitivity at 1 month of age for perinatally infected infants was 89%^8^
- in formula-fed infants who received 6 weeks of postpartum zidovudine (AZT), with or without other antiretrovirals, 32% of intrapartum-infected infants tested HIV DNA PCR negative at 6 weeks of age but tested positive at 3 months of age^9^
- prophylaxis reduces HIV DNA concentrations at birth complicating early identification of infected infants for initiation of early treatment^10^
- there are case studies illustrating the challenges of ‘false negative’ and ‘indeterminate’ HIV PCR results in early infant diagnosis in the context of current PMTCT prophylaxis and calling for revised diagnostic guidelines.^11,12^

The 6-week test, conveniently scheduled at the same time as the 6-week expanded programme for immunisation (EPI) visit, is therefore too late and too early to detect all *in utero* and intrapartum HIV infections in the context of current PMTCT interventions. It is too late to diagnose infants who die prior to 6 weeks of age and to achieve early combination antiretroviral therapy (cART) initiation by 7.4 weeks of age as was done on the Children with HIV Early Antiretroviral Therapy (CHER) trial to reduce early morbidity and mortality.^13^ It is too early to diagnose *in utero* and intrapartum infections suppressed by daily dose NVP and/or maternal prophylaxis via the placenta and/or breastmilk.

## Diagnostic performance of HIV PCR at birth

The HIV PCR sensitivity at birth for detecting perinatal HIV infections (viz. *in utero* and intrapartum HIV infections) can never approach 100% because it detects *in utero* infection only and cannot detect intrapartum infection, namely an infection transmitted at the time of labour and delivery and essentially in the window period at birth. Prior to the implementation of PMTCT and the use of standardised HIV assays, Dunn et al.’s meta-analysis demonstrated that 38% of all perinatal infections were detectable at birth.^14^ With World Health Organization (WHO) Option A prophylaxis and a single dose of NVP at birth for infants, Lilian et al. demonstrated that 76% of all early HIV infections were detectable at birth.^15^ This increase was attributed to more sensitive viral detection assays and a proportional increase in *in utero* to intrapartum infections as a result of PMTCT prophylaxis targeting intrapartum infections during late pregnancy and delivery. As the majority of women deliver in health facilities in South Africa, identifying all HIV-infected women at delivery coupled with birth HIV PCR testing would yield three-quarters of all perinatal HIV infections with close to 90% coverage.

With Option B or B+ including daily dose NVP for 6 weeks to the infant, the ratio of *in utero* to intrapartum infections detectable at birth and 6 weeks of age is not known but is likely to be similar to Option A.

## When should birth and early neonatal HIV PCR be considered?

Because HIV testing of neonates is not performed unless a neonate is symptomatic, the HIV-related neonatal mortality rate in South Africa is unknown. In the presence of vertical transmission and without a birth diagnosis, it is neither possible to detect neonatal HIV infection nor to reduce HIV-related neonatal mortality by initiating antiretroviral therapy. Approximately 20% of infants known to be HIV-infected at birth in Johannesburg either died or were lost to follow-up by the time that 6-week HIV PCR testing was conducted.^4^

Modeling the ideal timing of HIV PCR tests in early infant diagnosis for South Africa,^16^ considering birth, 6-, 10- and 14-week EPI visits, demonstrated that:

- when using 1 HIV PCR test, the same number of HIV-infected infants are identified when testing at birth or at 6 weeks of age
- when using 2 HIV PCR tests, the most HIV-infected ­infants can be identified by testing at birth and 10 weeks of age.

Additional evidence for the assumptions made in the model would assist in refining the optimal timing of HIV PCR tests.

The 2014 South African National Consolidated Guidelines recommend targeted birth testing, namely HIV PCR testing of only those HIV-exposed neonates identified as being at high risk of HIV transmission.^1^ ‘High risk’ includes all premature (born before 37 weeks’ gestational age), low birth weight (LBW < 2500 g) or symptomatic HIV-exposed neonates and those born to women who were unbooked or received a late diagnosis of HIV (e.g. at delivery) or received < 4 weeks of antiretroviral PMTCT prophylaxis or had viral loads > 1000 copies per millilitre or were co-infected with tuberculosis during pregnancy. The Western Cape PMTCT guidelines also recommend targeted birth testing that include additional and slightly modified high-risk factors.^17^ These high-risk factors predict vertical transmission but not necessarily *in utero* transmission, and therefore the birth HIV PCR test may be negative.

Universal birth testing of all HIV-exposed infants may be simpler to implement than targeted birth testing. The cost of performing two early HIV PCR tests, to detect *in utero* and intrapartum infections, on every HIV-exposed infant can be offset by following 2010 WHO guidelines to use HIV rapid tests (HRT) from 9 months of age.^18^ As the majority of HIV-exposed, uninfected infants demonstrate seroreversion by 9 months of age by testing HRT negative, only those with positive HRT would require HIV PCR tests.^19^ This approach reduces the number of HIV PCR tests required for symptomatic infants or those requiring testing post-cessation of breastfeeding.

## Optimal response to a birth or early neonatal HIV PCR result that is positive

To avoid morbidity and mortality, all positive HIV PCR results require urgent action to (1) confirm the HIV-infected status on a second blood sample and (2) initiate cART. Healthcare facilities require good communication with the laboratory to access positive results within 2–7 days and systems for patient follow-up to see all HIV PCR-positive patients as soon as possible. As neonates usually return to their primary healthcare clinic and not the maternity unit for follow-up after birth, ensuring that birth HIV PCR test results reach patients will be pivotal to successful implementation of birth testing. Point of care (POC) HIV diagnosis would facilitate same-day identification of HIV-infected neonates.

The results of the second, confirmatory viral detection assay should not delay cART initiation but should be obtained as early as possible because, once cART is initiated, it becomes progressively more difficult to detect HIV either by HIV PCR or viral load testing. The availability of two different POC assays for detection of HIV would facilitate same-day identification and confirmation of HIV infection in neonates.

## Further testing if birth or early neonatal HIV PCR test result is negative

If the birth HIV PCR test is negative, an HIV PCR test at 10 weeks of age is recommended to detect as many cases of intrapartum infection as possible. If the HIV PCR test is repeated at 6 weeks of age, as per national guidelines,^1^ fewer cases of intrapartum infection may be identified owing to the viral load lowering effect of the daily dose NVP infant prophylaxis. The 2014 National Consolidated Guidelines cater for this in high-risk infants only by recommending a third HIV PCR at 16 weeks of age where prolonged infant prophylaxis (e.g. daily dose NVP for 12 weeks) has been used.^1^ The probability that the sensitivity of diagnostic virological assays is affected by antiretroviral prophylaxis has prompted American guidelines to recommend an additional HIV PCR test be performed 2–4 weeks after combination antiretroviral infant prophylaxis has been discontinued if negative HIV PCR results were obtained during prophylaxis.^20^ As for all HIV-exposed and uninfected infants, HIV PCR testing is recommended whenever clinical features suggestive of HIV infection are present and 6 weeks after cessation of breastfeeding (if < 18 months old). If, at 6 weeks after weaning, the child is ≥ 18 months old, a HRT or HIV enzyme-linked immunosorbent assay (ELISA) test should be performed instead of an HIV PCR test.

In an evolving PMTCT environment, ongoing monitoring is necessary to assess the impact of early diagnosis of HIV infection in neonates and to ensure that an evidence-based, effective diagnostic algorithm is deployed.
